# Exploring Components of the CO_2_-Concentrating Mechanism in Alkaliphilic Cyanobacteria Through Genome-Based Analysis

**DOI:** 10.1016/j.csbj.2017.05.001

**Published:** 2017-05-25

**Authors:** Amornpan Klanchui, Supapon Cheevadhanarak, Peerada Prommeenate, Asawin Meechai

**Affiliations:** a Biological Engineering Program, Faculty of Engineering, King Mongkut's University of Technology Thonburi, Bangkok 10140, Thailand; b Division of Biotechnology, School of Bioresources and Technology, King Mongkut's University of Technology Thonburi, Bangkok 10150, Thailand; c Biochemical Engineering and Pilot Plant Research and Development (BEC) Unit, National Center for Genetic Engineering and Biotechnology, National Science and Technology Development Agency at King Mongkut's University of Technology Thonburi, Bangkok 10150, Thailand; d Department of Chemical Engineering, Faculty of Engineering, King Mongkut's University of Technology Thonburi, Bangkok 10140, Thailand

**Keywords:** Inorganic carbon uptake, CO_2_-concentrating mechanism, Carbonic anhydrase, Carboxysomes, Alkaliphilic cyanobacteria, Genomic data

## Abstract

In cyanobacteria, the CO_2_-concentrating mechanism (CCM) is a vital biological process that provides effective photosynthetic CO_2_ fixation by elevating the CO_2_ level near the active site of Rubisco. This process enables the adaptation of cyanobacteria to various habitats, particularly in CO_2_-limited environments. Although CCM of freshwater and marine cyanobacteria are well studied, there is limited information on the CCM of cyanobacteria living under alkaline environments. Here, we aimed to explore the molecular components of CCM in 12 alkaliphilic cyanobacteria through genome-based analysis. These cyanobacteria included 6 moderate alkaliphiles; *Pleurocapsa* sp. PCC 7327, *Synechococcus* spp., *Cyanobacterium* spp., *Spirulina subsalsa* PCC 9445, and 6 strong alkaliphiles (i.e. *Arthrospira* spp.). The results showed that both groups belong to β-cyanobacteria based on β-carboxysome shell proteins with form 1B of Rubisco. They also contained standard genes, *ccmKLMNO* cluster, which is essential for β-carboxysome formation. Most strains did not have the high-affinity Na^+^/HCO_3_^−^ symporter SbtA and the medium-affinity ATP-dependent HCO_3_^−^ transporter BCT1. Specifically, all strong alkaliphiles appeared to lack BCT1. Beside the transport systems, carboxysomal β-CA, CcaA, was absent in all alkaliphiles, except for three moderate alkaliphiles: *Pleurocapsa* sp. PCC 7327, *Cyanobacteriumstranieri* PCC 7202, and *Spirulina subsalsa* PCC 9445. Furthermore, comparative analysis of the CCM components among freshwater, marine, and alkaliphilic β-cyanobacteria revealed that the basic molecular components of the CCM in the alkaliphilic cyanobacteria seemed to share more degrees of similarity with freshwater than marine cyanobacteria. These findings provide a relationship between the CCM components of cyanobacteria and their habitats.

## 1 Introduction

CO_2_-concentrating mechanism (CCM) is an important process that maximizes the efficiency of inorganic carbon (C_i_; CO_2_ and HCO_3_^−^) uptake and CO_2_ fixation in cyanobacteria and eukaryotic algae [1]. It elevates CO_2_ level near the active site of Ribulose-1,5-bisphosphate carboxylase/oxygenase (Rubisco) enclosed in a polyhedral microcompartment called carboxysomes, thus enhancing photosynthetic performance [2]. In cyanobacteria, CCM is the key process that enables them to adapt to their diverse ranges of CO_2_-limited aquatic environments such as freshwater, marine, and alkaline lakes [3], [4], [5]. Insights into the basic molecular components of cyanobacterial CCM in relation to their habitats may provide us with an efficient strategy for improvement of photosynthetic CO_2_ fixation and biomass yield in these organisms [6], [7] and crop plants [8], [9].

In general, the cyanobacterial CCM consists of two primary components –C_i_ uptake systems and carboxysomes– as described below.

### 1.1 C_i_ Uptake Systems

The C_i_ uptake systems are comprised of two uptake systems of CO_2_ [10], [11] and three transport systems of HCO_3_^−^ [12], [13]. The CO_2_ uptake systems, located at the thylakoid membrane, convert cytosolic CO_2_ into HCO_3_^−^ [14]. These systems are based on NAD(P)H dehydrogenase type 1 (NDH-1) complexes comprising of NDH-1_3_ and NDH-1_4_ protein complexes. NDH-1_3_ is the low-CO_2_ inducible high-affinity CO_2_ uptake system, encoded by *ndhD3*, *ndhF3*, and *cupA*(*chpY*). On the other hand, NDH-1_4_ protein complex is the constitutive low-affinity CO_2_ uptake system encoded by *ndhD4*, *ndhF4*, and *cupB*(*chpX*) genes [15]. While protein subunits NdhD and NdhF are responsible for CO_2_ uptake [10], CupA and CupB catalyze the hydration reaction of CO_2_ into HCO_3_^−^ [16]. For the transport of HCO_3_^−^_,_ it is facilitated by three transporters, located at the plasma membrane, including BicA (a SulP-type sodium dependent HCO_3_^−^ transporter), SbtA (a sodium-dependent HCO_3_^−^ symporter), and BCT1 (an ATP-binding cassette (ABC)-type HCO_3_^−^ transporter). These three transporters have different properties. BicA has low affinity for bicarbonate (K_m_ = 70–350 μM), but high flux of HCO_3_^−^ uptake, while SbtA has high affinity for bicarbonate (K_m_ < 5 μM), but low flux of HCO_3_^−^ uptake [12], [17]. BCT1 has medium substrate affinity for bicarbonate (K_m_ = 10–15 μM) and low flux of HCO_3_^−^ uptake [18]. The operation of the C_i_ uptake systems ends up with a cytosolic C_i_ pool in the form of HCO_3_^−^, which is subsequently diffused into carboxysomes.

### 1.2 Carboxysomes

Carboxysomes are specialized sub-cellular compartments composing of protein shells and two encapsulated enzymes, Rubisco and carbonic anhydrase (CA) [19], [20]. In carboxysomes, CA catalyzes HCO_3_^−^ into CO_2_, which is a substrate for Rubisco [21]. There are two types of carboxysomes, α- and β-. The *cso*-type of shell proteins, encoded by *cso* operon, is termed α-carboxysomes, while the *ccm*-type of shell polypeptides, encoded by *ccmKLMNO* operon, is termed β-carboxysomes. Based on this criterion, the cyanobacterial species carrying form 1A of Rubisco within α-carboxysomes are classified as α-cyanobacteria while the species containing form 1B of Rubisco within β-carboxysomes are classified as β-cyanobacteria [22], [23]. Although the two carboxysome types are different in gene organization, formation, and species distribution, they have similar functions which are to limit CO_2_ leaking, reduce the risk of photorespiration, and enhance the carboxylase activity of Rubisco [20], [24]. Among the β-carboxysome proteins, which have been extensively studied, CcmK, CcmL, and CcmO were proposed to be in the outer shell layer [25], [26], while CcmM and CcmN were proposed to localize in the inner shell [27]. Concerning on CA, various carboxysomal CA have been reported. They are named β-CA (CcaA) and γ-CA (CcmM) in β-cyanobacteria [28] and named β-CA (CsoSCA) in α-cyanobacteria [29]. β-cyanobacterial species also contain two types of non-carboxysomal CAs, β-CA (EcaB) and α-CA (EcaA), localized in the cell membrane or in the periplasmic space [30]. However, the specific function of EcaA/B has not yet been confirmed. For Rubisco, it catalyzes CO_2_ fixation reaction to generate 3-phosphoglycerate as a precursor for the Calvin-Benson-Bassham cycle. This enzyme consists of eight small (RbcS; 12-18 kDa) and eight large (RbcL, 50–55 kDa) subunits [31]. Assembly of Rubisco requires chaperone proteins [32]. RbcX encoded by *rbcX* is a Rubisco assembly chaperone, which interacts with RbcL to facilitate the assembly of RbcL and RbcS to form Rubisco holoenzyme [33], [34]. It has been reported that RbcX is highly conserved in organisms having form 1B Rubisco [35].

Cyanobacteria tend to have different sets of CCM components depending on their habitats [1]. Studies have shown that α- and β-cyanobacteria occupy different environments [36]. Most of the α-cyanobacteria such as *Prochlorococcus* and *Synechococcus* strains inhabit marine while β-cyanobacteria such as *Synechocystis* sp. PCC 6803 [37], [38], [39], *Anabaena variabilis* [40], and *Synechococcus elongatus* PCC 7942 [41] live mainly in freshwater. The two distinct environments differ mainly in their conditions such as pH, C_i_ content, and salinity. The factor that affects CCM the most is pH because it is strongly linked to the equilibrium of C_i_ species (H_2_CO_3_, CO_2_, HCO_3_^−^, and CO_3_^2 −^) in a system [42]. At high pH (> 9), C_i_ content is usually high with dominant CO_3_^2 −^ and HCO_3_^−^ ions, while pH 6–8, HCO_3_^−^ is mostly present. At low pH (< 6), C_i_ content is low with dominant CO_2_ and H_2_CO_3_ ions. It is reported that the C_i_ concentration in marine environment (pH ≈ 8.2) is fairly constant around 2 mM [3]. The C_i_ availability in freshwater (pH ≈ 7) is however lower and fluctuates [43]. Based on C_i_ content, freshwater cyanobacteria tend to have complete C_i_ uptake systems which allow them to cope with the C_i_ fluctuation, whereas marine strains appear to lack some C_i_ uptake systems because they mainly experience with stable environment [3]. In addition, some cyanobacteria can also survive in alkaline environments (pH = 8.5–11) [44]. An example of alkaline environments is soda lake which is characterized by the strong alkaline (pH ≥ 9.5) and high C_i_ concentration dominated with HCO_3_^−^ and CO_3_^2 −^ ions [43]. Although CCM of freshwater and marine cyanobacteria are well studied, only a few observations of CCM in alkaliphilic cyanobacteria have been reported [43], [45]. Some researchers have hypothesized that CCM might not be necessary in the alkaliphilic cyanobacteria because of unlimited supply of inorganic carbon in the form of HCO_3_^−^ and CO_3_^2 −^ in the alkaline environments. Nevertheless, CCM components have recently been identified in some alkaliphilic cyanobacteria. In 2007, Dudoladova et al. [46] discovered α- and β-classes of CA and their sub-cellular localization in *Rhabdoderma lineare*. Later, Mikhodyuk et al. [47] studied the transport systems for carbonate of the natronophilic cyanobacterium *Euhalothece* sp. Z-M001. Recently, with the availability of a complete genome sequence of *Microcoleus* sp. IPPAS B-353, Kupriyanova et al. [48] identified a whole set of putative CCM components in this alkaliphilic organism living in soda lakes. They found that composition of the CCM components of the *Microcoleus* strain is similar to that of *Synechocystis* sp. PCC 6803 and *Synechococcus* PCC 7002 which are freshwater and marine β-cyanobacteria, respectively.

To further explore alkaliphilic cyanobacterial CCM, we aimed to probe unique features of molecular components of CCM in 12 alkaliphilic strains and relationship with their habitat. All the candidate genes/proteins involved in C_i_ uptake systems and carboxysomes of 12 alkaliphilic strains, including those inhabiting moderate (pH 8.5–9.4) and strong alkaliphilic (pH ≥ 9.5) environments, were identified. Computational identification of orthologous proteins was performed between the selected alkaliphiles and the ‘model’ β-cyanobacterium, *Synechocystis* sp. PCC 6803, whose CCM has been well studied. By sequence-based analysis, the variation of CCM components and potential orthologous sequences associated with such components was proposed. Comparative analyses within alkaliphilic, freshwater, and marine β-cyanobacteria were investigated, and the relationship between CCM components and ecological adaptation of alkaliphilic cyanobacteria were also emphasized. Since CCM is the crucial mechanism for CO_2_ fixation and photosynthesis in cyanobacteria, we believe that a better understanding of the CCM components could pave the way for future research towards cellular improvement of economically important cyanobacteria such as *Arthrospira* spp.

## 2 Materials and Methods

### 2.1 Protein Sequence Retrieval

Amino acid sequences of 27 proteins involved in CCM of β-cyanobacterium, *Synechocystis* sp. PCC 6803 (Assembly ID GCA_000009725.1), were retrieved from the CyanoBase (http://genome.kazusa.or.jp/cyanobase) as a reference target protein set. These proteins were NdhD4 (gene ID *sll0027*), NdhF4 (gene ID *sll0026*), CupB (gene ID *slr1302*), NdhD3 (gene ID *sll1733*), NdhF3 (gene ID *sll1732*), CupA (gene ID *sll1734*), BicA1 (gene ID *sll0834*), BicA2 (gene ID *slr0096*), SbtA (gene ID *slr1512*), SbtB (gene ID *slr1513*), CmpA (gene ID *slr0040*), CmpB (gene ID *slr0041*), CmpC (gene ID *slr0043*), CmpD (gene ID *slr0044*), CcmK1 (gene ID *sll1029*), CcmK2 (gene ID *sll1028*), CcmK3 (gene ID *slr1838*), CcmK4 (gene ID *slr1839*), CcmL (gene ID *sll1030*), CcmM (gene ID *sll1031*), CcmN (gene ID *sll1032*), CcmO (gene ID *slr0436*), RbcL (gene ID *slr0009*), RbcS (gene ID *slr0012*), RbcX (gene ID *slr0011*), CcaA (gene ID *slr1347*), and EcaB (gene ID *slr0051*).

The genome, protein sequences, and annotation data of the 12 selected alkaliphiles were retrieved from the database of the National Center for Biotechnology Information (NCBI) in October, 2016. The analyzed alkaliphilic cyanobacteria included *Pleurocapsa* sp. PCC 7327 (P7; GenBank CP003590) [49], *Synechococcus* sp. JA-2-3B′a(2–13) (S2; GenBank NC_007776) [50], *Synechococcus* sp. JA-3-3Ab (S3; GenBank NC_007775) [50], *Cyanobacterium* PCC 7702 (CP; GenBank NZ_KB235926) [49], *Cyanobacterium stranieri* PCC 7202 (CS; GenBank CP003940) [49], *Spirulina subsalsa* PCC 9445 (SS; GenBank NZ_JH980292) [49], *Arthrospira platensis* C1 (AC; GenBank NZ_CM001632) [51], *Arthrospira platensis* NIES-39 (AN; GenBank NC_016640) [52], *Arthrospira platensis* str. Paraca (AP; GenBank ACSK00000000) [53], *Arthrospira maxima* CS-328 (AM; GenBank ABYK00000000) [54], *Arthrospira* sp. PCC 8005 (A8; GenBank NZ_FO818640) [55], and *Arthrospira* sp. TJSD091 (AT; GenBank LAYT00000000) [56].

### 2.2 Identification of Orthologous Proteins

The bidirectional sequence alignment approach, namely reciprocal BLASTP [57], was employed to identify proteins of 12 studied species, which are homologous to the reference proteins of *Synechocystis* sp. PCC 6803. To avoid under- and over-estimation of sequence similarity of these related species, the candidate orthologous proteins were determined based on BLAST statistics with the E-value threshold (≤ 10^− 6^) [58], the identity (≥ 30) [58], and coverage percentage (≥ 60) [58]. Only protein sequences with the BLASTP scores above the set critical values were further analyzed for the conserved domain using the Pfam database 27.0, provided by the Sanger Centre, UK (http://pfam.xfam.org/search) [59]. The default E-value cut-off of 1.0 was applied for this study [60]. The GUIDANCE web-server tool (http://guidance.tau.ac.il/) [61] was used to evaluate a confidence score of multiple sequence alignments. Additionally, the genomic features were visualized by GView [62].

### 2.3 Phylogenetic Analysis

A phylogenetic tree of the 12 selected strains and reference cyanobacteria was constructed based on Rubisco large subunit (RbcL) amino acid sequences, which were used to infer the protein function and classification among the strains. Other phylogenetic trees based on protein sequences of CmpABCD of the HCO_3_^−^ transporter BCT1 and sequences of NrtABCD of the nitrite/nitrate transporter were constructed to confirm the identity between the proteins. The reference species were selected according to types of carboxysomes (α- and β-classes), the existence of both CmpABCD and NrtABCD transporters in genomes, or their habitats. These strains included freshwater (*Anabaena* sp. PCC 7120, *Anabaena variabilis* ATCC 29413, *Cyanothece* sp. PCC 8801, *Cyanothece* sp. PCC 8802, *Nostoc punctiforme* ATCC 29133, *Synechococcus* sp. PCC 7942, and *Synechocystis* sp. PCC 6803) and marine (*Lyngbya* sp. PCC 8106, *Trichodesmium erythraeum* IMS101, *Synechococcus* sp. PCC 7002, *Synechococcus* sp. CC9605, *Synechococcus* sp. CC9902, *Prochlorococcus marinus* AS9601, NATL1A, and NATL2A, and *Prochlorococcus marinus* MIT 9211, 9215, 9301, 9303, 9312, 9313, and 9515) cyanobacteria. Their corresponding amino acid sequences were retrieved from the public databases, including the CyanoBase (http://genome.kazusa.or.jp/cyanobase) and the GenBank (http://www.ncbi.nlm.nih.gov/genbank) databases. A phylogenetic tree was created by performing multiple sequence alignment with MUSCLE [63], [64], and then constructed based on the Maximum Likelihood [65] through the MEGA 6.0 software [66]. The reliability of the trees/branches was estimated via the bootstrap method [67], with 3000 replications.

## 3 Results

### 3.1 Strains and Classification of Alkaliphilic Cyanobacterial CCM

In this study, 12 selected alkaliphilic cyanobacterial strains were defined based on their ability to grow in an alkaline environment (pH roughly 8.5–11). The chosen strains included both unicellular and filamentous blue-green algae, which have different original habitats. According to the habitat pH values, we classified the selected strains into two main groups: moderately alkaliphilic cyanobacteria (pH 8.5–9.4) and strongly alkaline cyanobacteria (pH ≥ 9.5) (Table 1). The first group, moderately alkaline cyanobacteria, was comprised of two subgroups: alkali-thermophile and alkali-mesophile. The subgroup alkali-thermophile consisted of four species isolated from alkaline hot spring environments (pH 8.5–8.8, 50–70 °C), P7, S2, S3, and CP. The alkali-mesophile group of cyanobacteria was composed of two euryhaline cyanobacteria, CS and SS, living under a saline and alkaline environment (pH 8.5–8.8, 30–45 °C). The strongly alkaline cyanobacteria group was comprised of six *Arthrospira* strains (AC, AN, AP, AM, A8, and AT), a dominant genus found in natural soda lakes, with growth optimum at pH range of 9.5–10.5 [68], [69]. More details about the ecological niches of all studied alkaliphilic strains are given in Table 1.

**Table 1:** Ecological niches of selected alkaliphilic cyanobacteria whose genome sequences are available (October 2016).

Species	Isolation site	Characteristics and habitats	Classification	Genome status	Reference
*Pleurocapsa* sp. PCC 7327 (P7)	Hunters hot spring, Oregon, USA	A unicellular nitrogen-fixing cyanobacterium. It is a stenohaline strain that can only survive within a narrow range of salinities. It has been found in alkaline water, hot spring (53 °C pH 8.5).	Moderate alkali-thermophile	Finished	[49]
*Synechococcus* sp. JA-2-3B′a(2–13) (S2) *Synechococcus* sp. JA-3-3Ab (S3)	Octopus Spring, Yellowstone National Park	A group of small (2–5 μm) unicellular cyanobacteria. Non-nitrogen-fixing cyanobacteria. The strain is dominant in alkaline siliceous hot springs (50–70 °C pH 8.5).	Moderate alkali-thermophile	Finished	[50]
*Cyanobacterium* PCC 7702 (CP)	Alkaline hot spring, near Reykjavik, Iceland	A practically unicellular cyanobacterium. The strain is isolated from alkaline siliceous hot springs (50–70 °C pH 8.8). Nitrogen-fixing and non-motile.	Moderate alkali-thermophile	Permanent Draft	[49]
*Cyanobacterium stranieri* PCC 7202 (CS)	Alkaline pond, Chad	A unicellular non-nitrogen-fixing cyanobacterium capable of growth in both freshwater and seawater media. Thus, it is able to adapt to a wide range of salinities (euryhaline). Non-motile.	Moderate alkali-mesophile	Finished	[49]
*Spirulina subsalsa* PCC 9445 (SS)	Alkaline-saline volcanic lake, Pantelleria, Italy	A motile filamentous cyanobacterium. It is capable of growth under a saline and alkaline environment.	Moderate alkali-mesophile	Permanent draft	[49]
*Arthrospira platensis* C1 (AC) *Arthrospira platensis* NIES-39 (AN) *Arthrospira platensis* str. Paraca (AP) *Arthrospira maxima* CS-328 (AM) *Arthrospira* sp. PCC 8005 (A8) *Arthrospira* sp. TJSD091 (AT)	Unknown Lake Chad, Chad, East Africa Unknown Lake Chad, Chad, East Africa Seaside wetland, China, Bohai Unknown	A group of filamentous cyanobacteria that have an important role in industrial applications. Non-heterocyst-forming and non-nitrogen-fixing cyanobacteria; hydrogen-producing strains. They grow naturally in a high-salt alkaline (carbonate/bicarbonate) open pond system. The optimum pH for growth of ordinary *Arthrospira* strains is in the range of 9.0–9.5. AC is a laboratory strain.	Strong alkaliphile	Permanent draft Permanent draft Permanent draft Permanent draft Permanent draft Permanent draft	[51], [52], [53], [54], [55], [56]

**Fig. 1:**
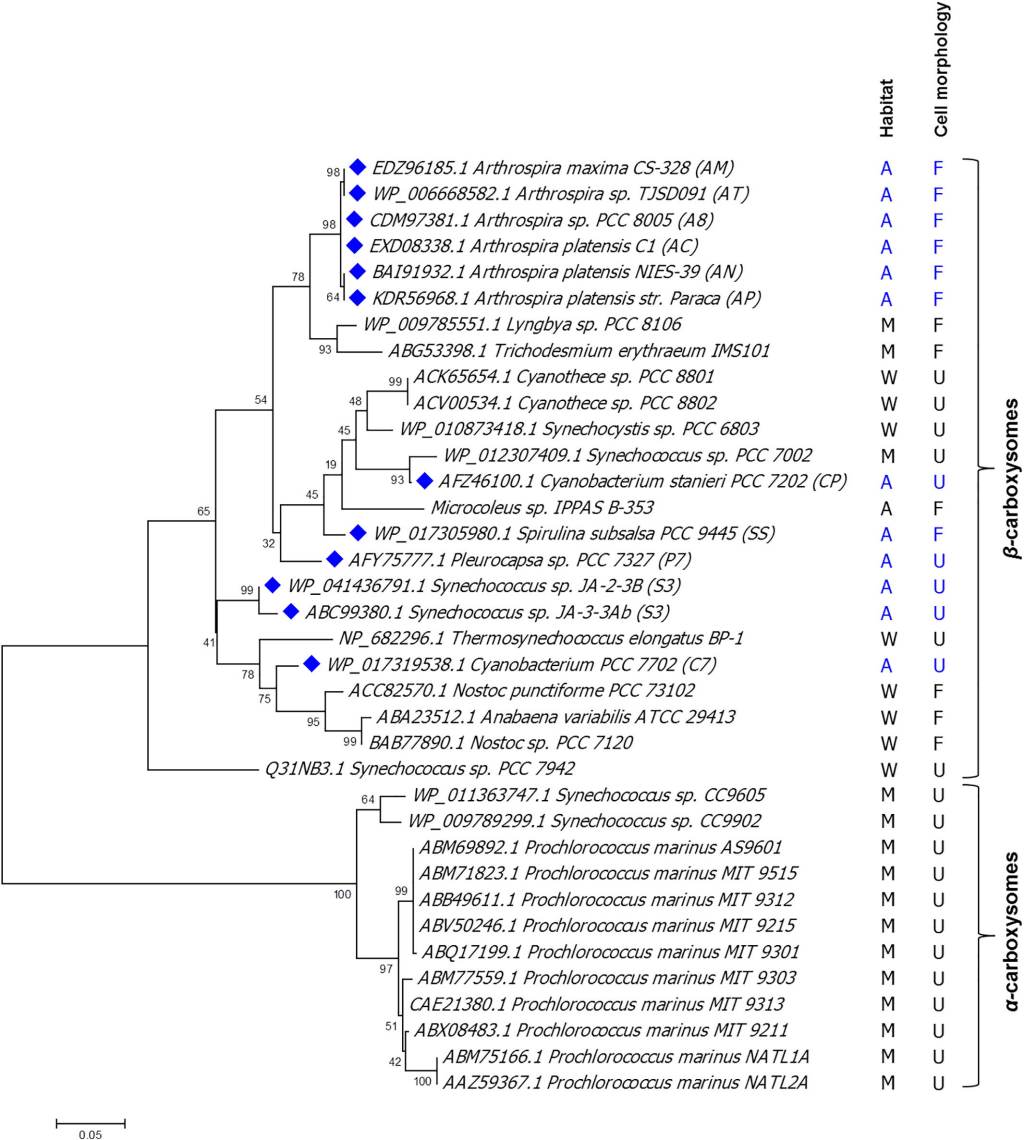
Phylogenetic tree based on Rubisco large subunit protein sequences. The 12 alkaliphilic cyanobacterial strains examined in this study are identified by the blue diamond, while other reference species are represented without diamond. Cyanobacterial habitat, cell arrangement, and carboxysome type (α- or β-) are displayed. Within the column for habitat, freshwater strains are denoted by W, marine by M, and alkaline niche by A. Unicellular and filamentous cell arrangement is represented by U and F, respectively.

To examine the carboxysome type operating in the 12 investigated cyanobacteria, a phylogenetic tree was constructed based on RbcL amino acid sequences. RbcL was chosen because it is a well-conserved enzyme for CO_2_ fixation and has been used for the classification of cyanobacteria groups before [70]. A total of 36 protein sequences from 36 cyanobacteria were analyzed; 12 chosen alkaliphilic cyanobacteria and 24 reference species consisting of 8 freshwater, 15 marine, and a haloalkaliphilic species. The results showed that all 36 cyanobacterial strains were divided into two main groups, β- and α-cyanobacteria, according to their Rubisco forms (Fig. 1). All 12 studied alkaliphilic cyanobacteria were clustered together in the β-cyanobacteria branch, reflecting the presence of the Rubisco 1B form. However, they were not completely grouped in the same cluster, following neither to their habitat nor morphology. For instance, four moderately alkali-thermophilic cyanobacteria, P7, S2, S3, and CP, were located in different clusters. In addition, SS which is a filamentous cyanobacterium appeared to be in the same cluster with the unicellular species.

### 3.2 Identification of Orthologous Proteins and Genes in Alkaliphilic Strains

Proteins corresponding to the CCM components of the 12 chosen alkaliphilic strains were identified as described in the Materials and Methods. All identified proteins had different degree of identity (40–80%, E-value threshold of ≤ 10^− 20^) with the reference sequences. They also contained conserved domain regions which were similar to the reference proteins. Annotation details, including gene and protein accession number, annotation scores, protein domain analysis, are available in Supplementary File. The presence and absence of genes encoding the CCM components of alkaliphilic strains are shown in Table 2. The comparison of molecular CCM components from the *Synechocystis* sp. PCC 6803 and from the analyzed species revealed that approximately 20 orthologous genes are present in the investigated alkaliphiles. Furthermore, the results showed that the moderately alkaline group possesses more CCM components than the strongly alkaline ones.

**Table 2:** Variation of the genes involved in CO_2_-concentrating mechanism among alkaliphilic cyanobacterial strains. *Pleurocapsa* sp. PCC 7327 (P7), *Synechococcus* sp. JA-2-3B′a(2–13) (S2), *Synechococcus* sp. JA-3-3Ab (S3), *Cyanobacterium* PCC 7702 (CP), *Cyanobacterium stranieri* PCC 7202 (CS), *Spirulina subsalsa* PCC 9445 (SS), *A. platensis* C1 (AC), *A. platensis* NIES-39 (AN), *A. platensis* Paraca (AP), *A. maxima* CS-328 (AM), *Arthrospira* sp. PCC 8005 (A8), and *Arthrospira* sp. TJSD091 (AT) Numbers represent copy number of genes; nf is referred to not found; genes coding for putative NrtABCD are denoted by the symbol “?”.

Component			Gene	Alkaliphilic strains											
				Moderate alkaliphilic cyanobacteria						Strong alkaliphilic cyanobacteria					
				Thermophiles				Mesophiles							
				P7	S2	S3	CP	CS	SS	AC	AN	AP	AM	A8	AT
C_i_ uptake systems	CO_2_ uptake	NDH-1_4_ complex	*ndhF4*	1	1	1	1	1	1	1	1	1	1	1	1
			*ndhD4*	1	1	1	1	1	1	1	1	1	1	1	1
			*cupB*	1	1	1	1	1	1	1	1	1	1	1	1
		NDH-1_3_ complex	*ndhF3*	1	1	1	1	1	1	1	1	1	1	1	1
			*ndhD3*	1	1	1	1	1	1	1	1	1	1	1	1
			*cupA*	1	1	1	1	1	1	1	1	1	1	1	1
	HCO_3_^−^ transport	BicA	*bicA1*	1	1	1	1	1	1	1	1	1	1	1	1
			*bicA2*	nf	nf	nf	nf	nf	1	1	1	1	1	1	nf
		SbtA SbtA regulator	*sbtA*	nf	nf	nf	nf	nf	1	nf	1	1	nf	nf	nf
			*sbtB*	nf	nf	nf	nf	nf	1	nf	1	1	nf	nf	nf
		BCT1	*cmpA*	1	1	1	1	?	?	?	?	?	?	?	?
			*cmpB*	1	1	1	1	?	?	?	?	?	?	?	?
			*cmpC*	1	1	1	1	?	?	?	?	?	?	?	?
			*cmpD*	1	1	1	1	?	?	?	?	?	?	?	?
Carboxysomes	Shell proteins	β-Carboxysomal shell proteins	*ccmK1*	1	1	1	1	1	1	1	1	1	1	1	1
			*ccmK2*	1	1	1	1	1	1	1	1	1	1	1	1
			*ccmK3*	1	nf	nf	1	1	1	1	1	1	1	1	1
			*ccmK4*	1	nf	nf	1	1	1	1	1	1	1	1	1
			*ccmL*	1	1	1	1	1	1	1	1	1	1	1	1
			*ccmM* (containing γ-CA domain)	1	1	1	1	1	1	1	1	1	1	1	1
			*ccmN*	1	1	1	1	1	1	1	1	1	1	1	1
			*ccmO*	1	1	1	1	1	1	1	1	1	1	1	1
	Encapsulated enzymes	Rubisco	*rbcL*	1	1	1	1	1	1	1	1	1	1	1	1
			*rbcS*	1	1	1	1	1	1	1	1	1	1	1	1
		β-CA	*ccaA*	1	nf	nf	nf	1	1	nf	nf	nf	nf	nf	nf

**Fig. 2:**
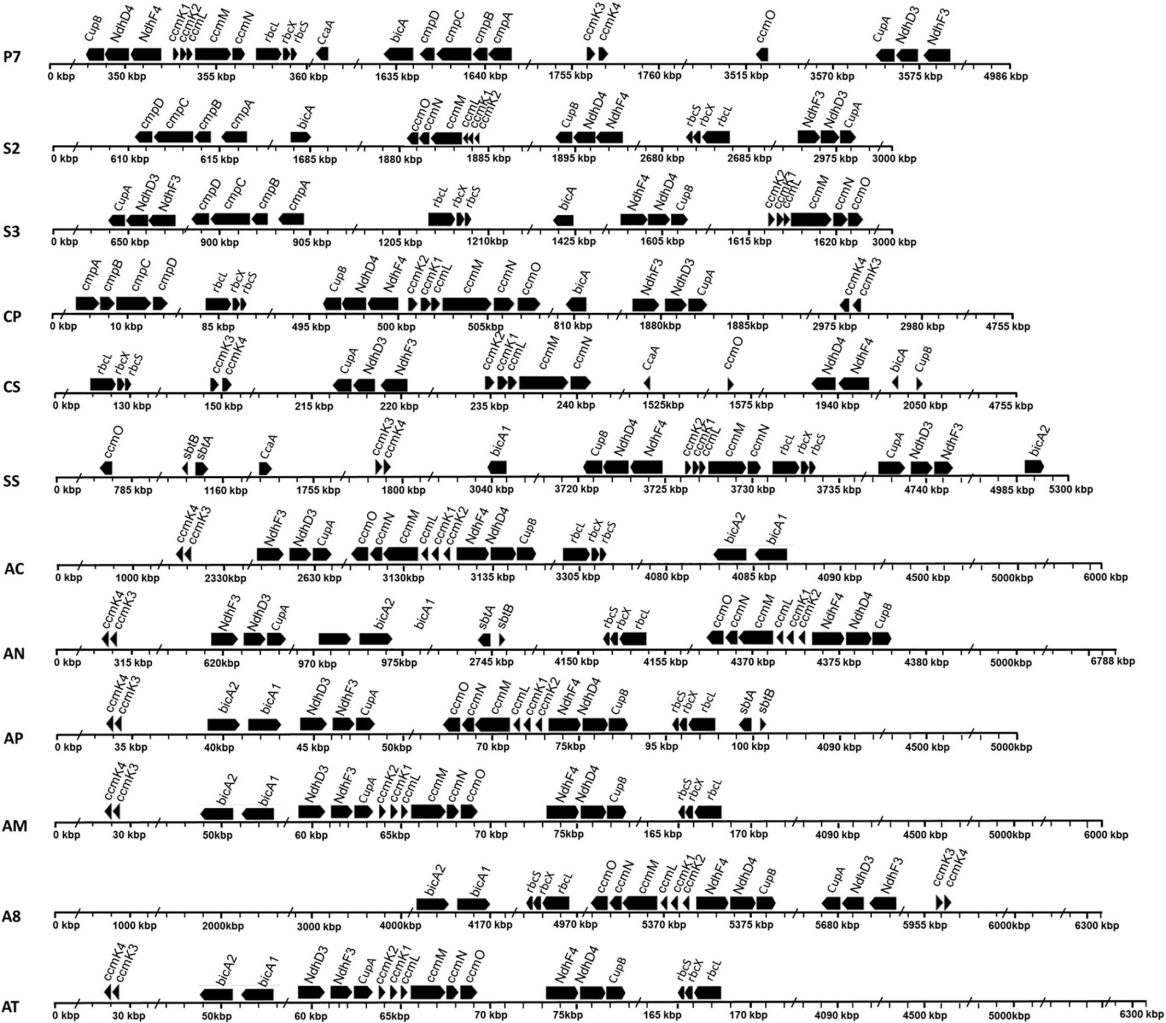
Comparative genomic structure and gene organization of the CCM in all 12 alkaliphilic cyanobacteria. Solid arrow boxes indicate genes and the direction of transcription. The completed genome sequence was available for P7, S2, S3, and CS, while for other strains there is only permanent draft genome information.

**Fig. 3:**
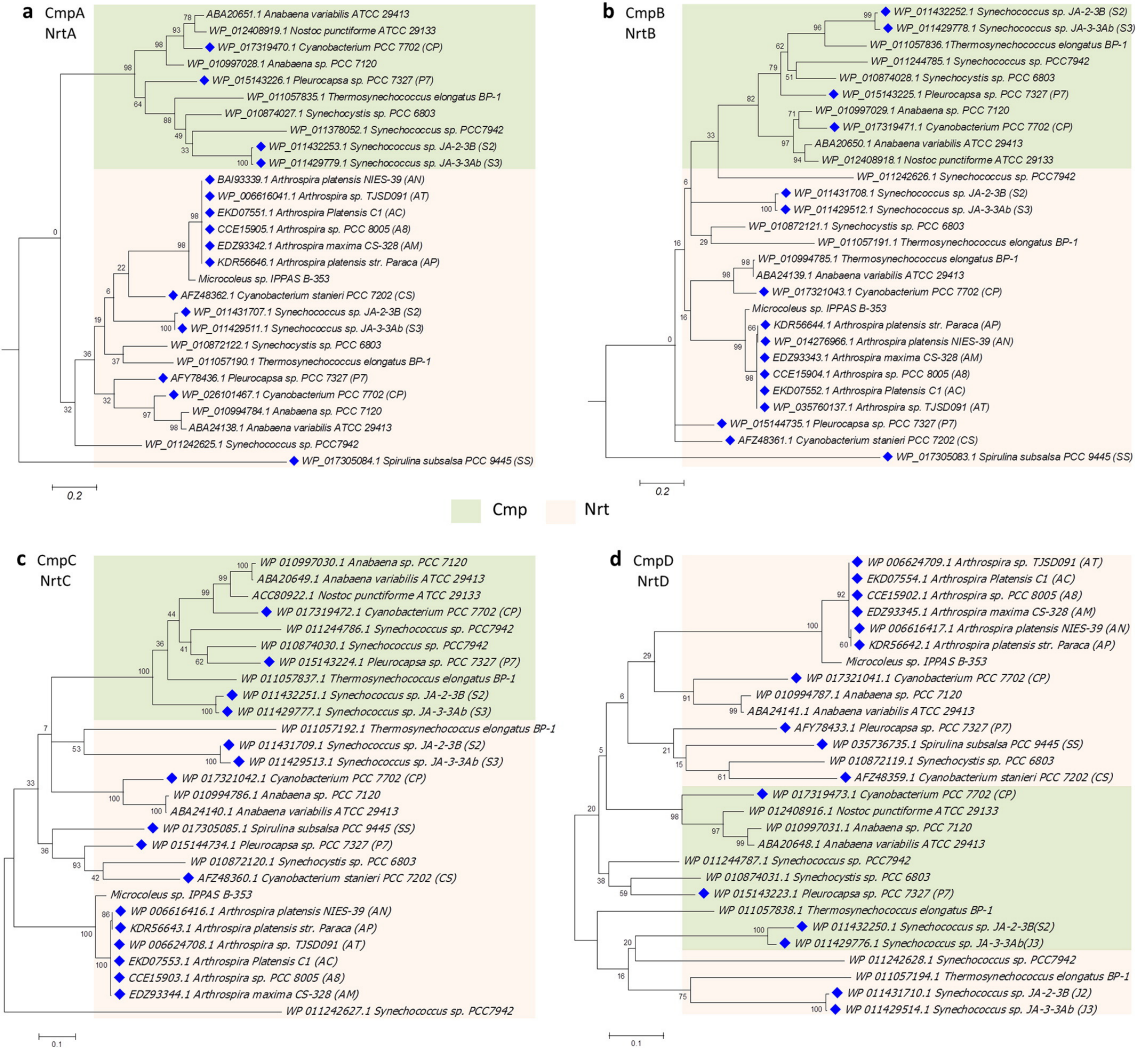
Phylogenetic trees of the CmpABCD proteins and NrtABCD proteins of the selected alkaliphilic cyanobacteria. The outgroup cyanobacteria includes *Synechococcus* sp. PCC 7942, which has an experimental study of HCO_3_^−^ transporter BCT1 and nitrite/nitrate transport system, NRT [12]. (a) Phylogenetic tree based on the CmpA and NrtA protein sequences. (b) Phylogenetic tree based on the CmpB and NrtB protein sequences. (c) Phylogenetic tree based on the CmpC and NrtC protein sequences. (d) Phylogenetic tree based on the CmpD and NrtD protein sequences. The alkaliphilic cyanobacteria are identified by the blue diamond, respectively. Cmp and Nrt families are highlighted in orange and green, respectively. Bootstrap values with 3000 replicates are shown at the nodes of the tree. The scale bars indicate the number of nucleotide substitutions per site.

Focusing on the C_i_ transport systems, there were up to five systems identified in the 12 alkaliphilic cyanobacteria: i) a low-affinity NDH-1_4_ complex (NdhD4/NdhF4/CupB); ii) a high-affinity NDH-1_3_ complex (NdhD3/NdhF3/CupA); iii) a SulP-type low-affinity Na^+^-dependent HCO_3_^−^ BicA transporter; iv) a high-affinity Na^+^/HCO_3_^−^ symporter SbtA; and v) a high-affinity ATP-binding cassette BCT1(CmpABCD). All protein sequences of NDH-1_4_ and NDH-1_3_ showed a high sequence similarity with the reference sequences. Genes encoding each NDH-1 complex were localized together (Fig. 2). All studied strains showed high degree of homology with the BicA of *Synechocystis* sp. PCC 6803 (≥ 60% of amino acid identity). However, the orthologs of the SbtA transporter were found only in SS, AN, and AP. *SbtB* gene encoding SbtB protein that possibly functions as SbtA regulator [71] was also found nearby *sbtA* in the opposite direction in these three strains (Fig. 2). For the third HCO_3_^−^ transporter, BCT1, the orthologs of CmpABCD and NrtABCD (nitrate/nitrite transport system) cluster were observed in all studied alkaliphiles. They exhibited a moderate sequence similarity (55–71%) with the reference proteins. Both CmpABCD and NrtABCD protein sequences contained a similar protein domain, PBP2_NrtA_CpmA. In addition, a confidence score of multiple sequence alignments from the GUIDANCE web-server tool (http://guidance.tau.ac.il/) [61] showed highly conserved regions among these two protein clusters. Since the CmpABCD and NrtABCD protein sequences have been previously reported to share high similarity in sequences belonging to the same ABC transporter family [72], the experimentally confirmed proteins CmpABCD of *Synechococcus* sp. PCC 7942, were included in the subsequent analysis to verify the previous annotation. The BLAST's results showed a high homology between CmpABCD of *Synechococcus* sp. PCC 7942 and the sequences retrieved from four species, P7, S2, S3, and CP, with ~ 65–75% identity and an E-value of ~ 10^− 100^. Moreover, the sequences were further identified using the phylogenetic analysis (Fig. 3). The trees showed that the candidate sequences of CmpABCD of four species, P7, S2, S3, and CP, were clustered into the CmpABCD of *Synechococcus* sp. PCC 7942 and the other reference cyanobacteria, while the putative NrtABCD sequences of eight cyanobacteria, CS, SS, and six *Arthrospira* spp., were clustered into the NrtABCD of the reference cyanobacterium. As such, it is likely that BCT1 is present only in the 4 out of the 12 studied alkaliphilic cyanobacteria. Nevertheless, an experimental study of the specificity of BCT1 to a certain substrate should be further performed to clarify the function of putative protein subunits (CmpABCD) in these four alkaliphilic cyanobacteria.

According to the observed C_i_ uptake systems (Table 2), the 12 analyzed alkaliphilic strains could be divided into three genotypes: I) strains containing NDH-1_3_, NDH-1_4_, BicA and BCT1, II) strains containing NDH-1_3_, NDH-1_4_, and BicA, and III) strains containing NDH-1_3_, NDH-1_4_, BicA, and SbtA. While the moderate alkali-thermophiles possessed genotype I, the moderate alkali-mesophiles (euryhaline) and strong alkaliphiles seemed to possess either genotype II or III. These results revealed that all alkaliphilic strains shared the same CO_2_ uptake systems. However, their distinctions were observed by the presence of BCT1, a HCO_3_^−^ transporter. This transporter appeared to exist only in the moderate alkali-thermophiles, but absent in all moderate alkali-mesophiles and strong alkaliphiles.

From the organization of the CCM genes, genes encoding carboxysome shell proteins in all alkaliphiles, except P7 and SS, were found to arrange in a cluster, *ccmKLMNO*, consisting of *ccmK1*, *ccmK2*, *ccmL*, *ccmM*, *ccmN* and *ccmO* (Fig. 2). In addition, *ccmK3* and *ccmK4* were also found to be present in the 10 strains, but S2 and S3. The protein sequences shared significant moderate similarity with the reference sequences retrieved from the model organism. The observed maximal homology was around ~ 60–70% identity, with an E-value of ~ 10^− 50^ (see Supplementary File). Of these, weak homologs (~ 40% similarity) were found only for CcmN sequences. Protein domain analysis showed that CcmK1-K4 and CcmO contained bacterial microcompartment (BMC) domain (Pfam00936), whereas CcmL (BMC-P) contained ethanolamine utilization (EutN) domain (Pfam03319). Regarding to CcmN, although low similarity was observed, multiple protein sequence alignment among all examined cyanobacteria with *Synechococcus* sp. PCC 7942 revealed two functionally conserved distinct regions at N- and C-terminals, which were separated by a poorly conserved linker. These results supported the functions of CcmKLMNO as carboxysome shell proteins in alkaliphilic cyanobacteria. Beside the shell proteins, the amino acid sequences of Rubisco subunits, RbcL and RbcS, were moderately conserved (60% identity with the reference sequences) in their sequences; this was compared with the assembly chaperone RbcX protein (~ 45% identity with the reference sequences). The *rbcLSX* gene clusters were found to appear in all investigated genomes, located up- and down-streams of the *ccmKLMNO* cluster as shown in Fig. 2.

**Fig. 4:**
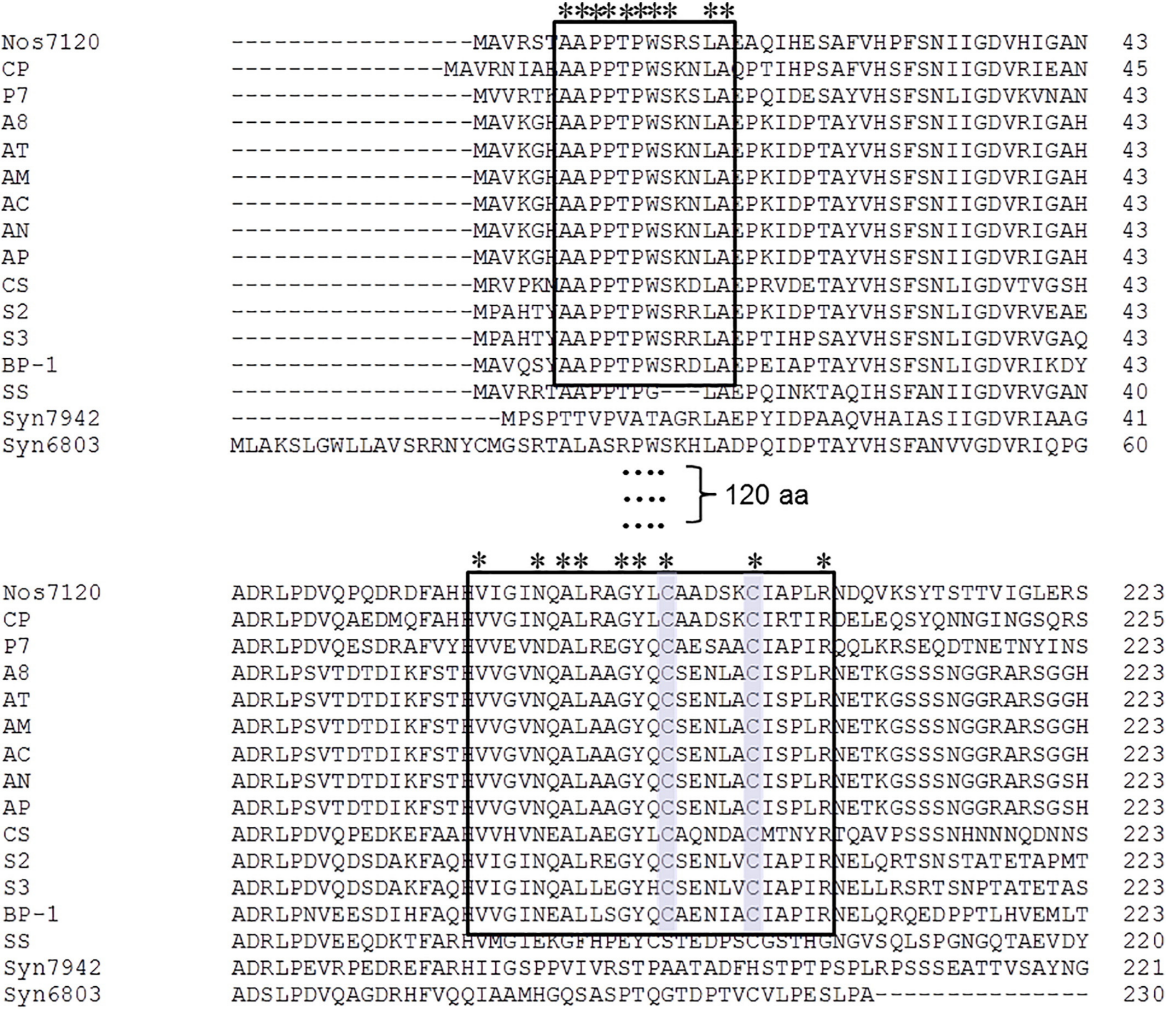
Partial alignment of CcmM amino acid sequences of 12 studied alkaliphiles with those of *Synechococcus elongatus* PCC 7942 (Syn7942), *Synechocystis* sp. PCC 6803 (Syn6803), *Thermosynechococcus elongatus* BP-1 (BP-1), and *Nostoc* sp. PCC 7120 (Noc7120) (GenBank accession no. BAA16773.1, Q03513.1, NP_681734, and BAB72822.1, respectively). The sequence order is based on the alignment. Boxes represent conserved regions of the N-terminal domain of CcmM that are assumed to be necessary for CA activity, according to Pena et al. [73]. Shaded cysteine amino acids showed essential residues participating in the disulfide bond in the C-termini of active CcmM protein. *Asterisks* indicate conserved amino acids inside such regions.

Focusing on β-CAs, which are enclosed in the carboxysome (CcaA) or localized in periplasmic space of β-cyanobacteria (EcaB), moderate similarity of amino acid sequence (~ 60% identity with reference protein) was found for CcaA proteins in only three studied cyanobacteria, P7, CS, and SS (Table 2). EcaB orthologs were not detected in any of the studied organisms. However, CcmM proteins of all alkaliphilic strains were found to have the γ-CA-like domain at N-terminal region. We further searched for other recognized CA classes in all 12 alkaliphilic cyanobacteria by using the protein sequences of α-CA, EcaA (*all2929*) of *Anabaena* sp. PCC 7120. The results showed no homologs sequences of EcaA in all 12 studied cyanobacteria. As a result, we concluded that all alkaliphilic species possessed γ-CA (CcmM), of which three moderate alkaliphiles contained additional β-CA (CcaA). To further evaluate a potential function of CcmM as an active CA, the comparative analysis of the γ-CA-like domain in N-terminal protein sequence was performed. The CcmM sequences from *Thermosynechococcus* *elongatus* BP-1 and *Nostoc* sp. PCC 7120 were included as functional γ-CA [73], [74]. The CcmM from *S. elongatus* PCC 7942 and *Synechocystis* sp. PCC 6803 were also included as a non-functional γ-CA [28], [75]. Fig. 4 shows the important amino acid residues in γ-CA-like domain of the 11 alkaliphilic strains, except SS, which were structurally similar to those of active CcmM in *T. elongatus* BP-1 [73] and *Nostoc* sp. PCC 7120 [74]. This result implied that the CcmM proteins of such 11 species might potentially have CA activity when the carboxysomal β-CA, CcaA, was missing.

### 3.3 Comparative Analysis of CCM Components Among β-Cyanobacteria

**Fig. 5:**
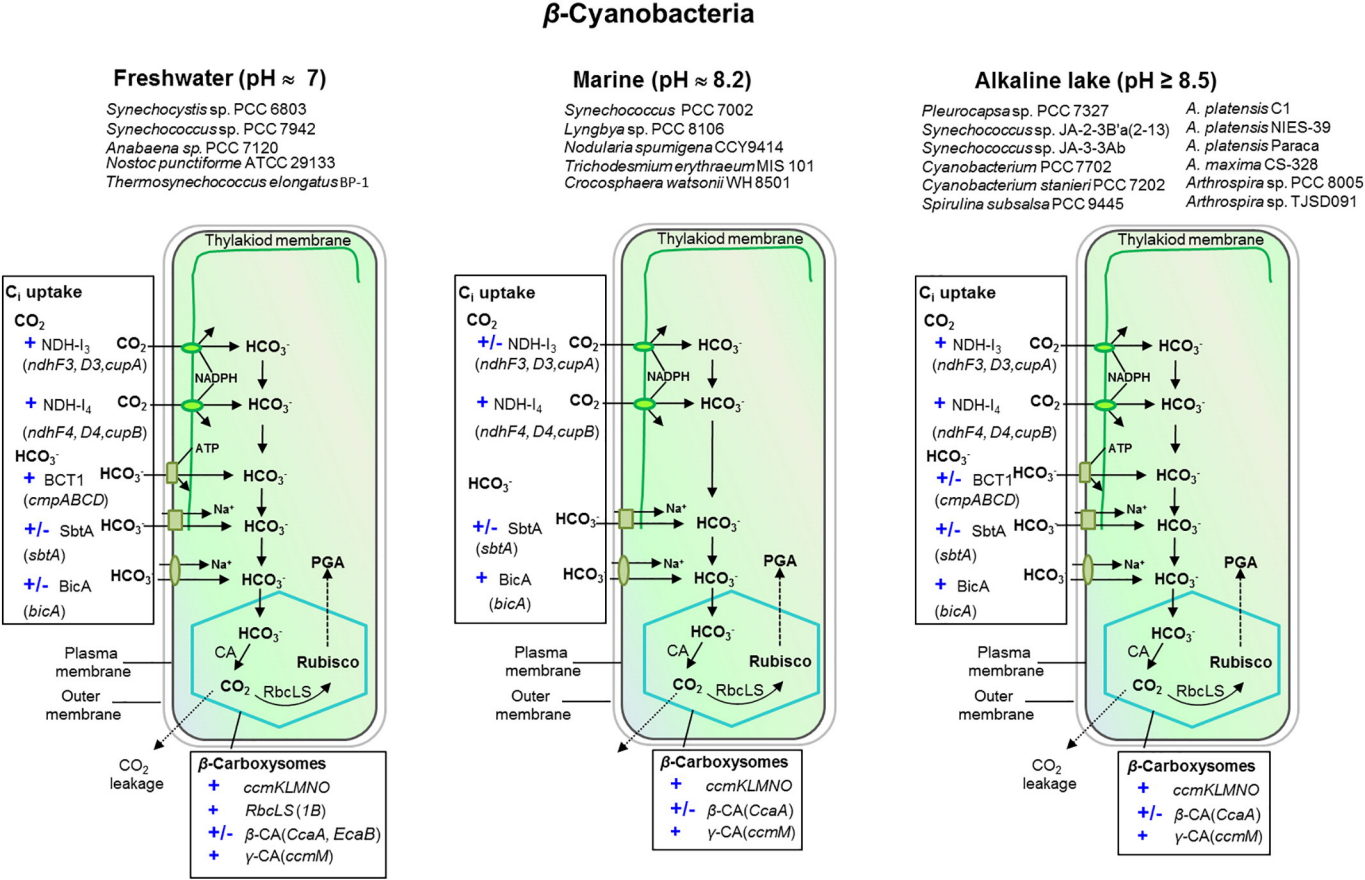
Diversity in characteristic components of the cyanobacterial CCM living in three different pH environments; freshwater (pH ~ 7), marine (pH ~ 8.2), and alkaline (pH > 8.5). The scheme is based on the literature data and is depicted for β-cyanobacteria. The species that were used to derive the groups are shown on the figure. CCM components of freshwater and marine cyanobacteria are adapted from [3]. CCM components of high alkaliphilic cyanobacterial type were identified in this study. + and ± indicate that the particular component is ‘always present’ and ‘sometimes present’, respectively. Designation: NDH-1_4_, low-affinity CO_2_ uptake system NDH-1_4_ complex; NDH-1_3_, low CO_2_-inducible high-affinity CO_2_ uptake system NDH-1_3_ complex; BCT1, ATP-binding cassette (ABC)-type high-affinity HCO_3_^−^ transporter; SbtA, high-affinity sodium-dependent HCO_3_^−^ symporter; BicA, SulP-type low-affinity sodium dependent HCO_3_^−^ transporter; CA, carbonic anhydrase; Rubisco, ribulose-1,5-bisphosphate carboxylase/oxygenase.

Comparative analysis of CCM components among β-cyanobacteria, living in freshwater (pH ~ 7), marine (pH ~ 8.2), and alkaliphilic (pH 8.5–11) strains were performed. Fig. 5 shows different CCM components among the three groups. The overall compositions of CCM components in alkaliphilic cyanobacteria were more similar to the freshwater than the marine groups. The cyanobacteria inhabiting freshwater and alkaline ecological niches possessed both CO_2_ uptake systems, NDH-1_3_ and NDH-1_4_, while most strains inhabiting marine habitats seemed to lack the NDH-1_3_. Focusing on the HCO_3_^−^ transport system, the results showed that marine and some alkaliphilic cyanobacteria consistently lacked the BCT1 type of the HCO_3_^−^ transporter. In addition, the freshwater β-cyanobacteria possessed the highest abundance of CAs, β-CA (CcaA and EcaB), α-CA (EcaA), and γ-CA (CcmM), while the alkaliphilic cyanobacteria were likely to possess only two conventional CAs, carboxysomal β-CA (CcaA) and γ-CA (CcmM). However, it should be noted that nine out of the twelve investigated alkaliphiles appeared to have only γ-CA (CcmM).

## 4 Discussion

Based on Rubisco phylogeny, all studied alkaliphilic cyanobacteria fall into β-cyanobacteria group (Fig. 1). It is obviously that the phylogeny of the studied alkaliphiles based on RbcL sequences can classify the cyanobacterial types; however, the tree is insufficient to elucidate the evolutionary relationship within the group based on cell morphology and habitats. Komarek et al. [76] previously performed a phylogenetic tree of 146 cyanobacterial OTUs using 31 conserved protein sequences and reported that the tree could not be clustered based on their morphology. Thus, phylogenetic analysis may not be an appropriate technique to unveil evolutionary relationship of cyanobacterial morphology and environments.

The presence of the *ccmKLMNO* cluster (Fig. 2) in all the 12 studied strains indicates the genes conserved in the *ccm* cluster of β-carboxysomes. Our finding suggests that all the investigated alkaliphilic cyanobacteria possess complete standard genes, which are essential for carboxysome formation. In addition, since CcmK3 and CcmK4 were considered as an accessory protein improving the functionality of the shell [26], the 10 studied strains found to possess CcmK1–4 would have a better shell protein function than the others. However, there is no obvious correlation between numbers of *ccmK* genes and environment niche (moderately to strongly alkaline habitat) of the examined strains.

Two systems of CO_2_ uptake, NDH-1_3_ and NDH-1_4_ complexes, were identified in all 12 analyzed strains (Fig. 5). Recently, Kupriyanova et al. [48] confirmed the presence of NDH-1_3_ and NDH-1_4_ in an alkaliphilic cyanobacterium *Microcoleus* sp. IPPAS B-353 and showed that genes corresponding to the NDH-1_3_ were transcribed and probably constitutively expressed. Both CO_2_ uptake systems were also observed in freshwater β-cyanobacteria and 20 strains of *M. aeruginosa* living in brackish waters and eutrophic lakes [77]. In contrast, the absence of NDH-1_3_ and/or NDH-1_4_ was reported in the oceanic α-cyanobacteria, *Prochlorococcus* species [36], and the marine β-cyanobacteria, *Trichodesmium erythraeum* species [3]. Thus, existence of NDH-1_3_ and NDH-1_4_ is apparently related to environments with varying CO_2_ availability. This is due to the distinct property of each complex in that NDH-I_3_ has a higher substrate affinity for CO_2_ than NDH-I_4_ [16]. The presence of both complexes in all 12 strains of alkaliphilic cyanobacterial seems to have an essential role in the survival and maintenance under CO_2_ fluctuation, particularly in alkaline environments (i.e. hot spring and soda lake).

Our finding for HCO_3_^−^ transport systems, BicA and SbtA, revealed that several alkaliphilic cyanobacteria strains have only one of the two transporters, preferably BicA. This may be due to the difference in their affinity for bicarbonate. BicA is a low-affinity and high flux rate bicarbonate transport system (K_m_ = 70–350 μM), while SbtA is high-affinity (K_m_ < 5 μM) HCO_3_^−^ transporter [17]. Possibly, the high-affinity SbtA is not necessary in most alkaliphilic strains typically inhabiting HCO_3_^−^ rich environment. If so, the organisms would most likely possess BicA rather than SbtA. Meanwhile, the presence of high-affinity transporter SbtA in SS, AN, and AP may, though indirectly, indicates that these organisms are able to face low concentrations of exogenous C_i_. Therefore, the presence of both BicA and SbtA in such strains might give a selective advantage in HCO_3_^−^ uptake and allow cell growth, enabling them to adapt in response to different C_i_ concentrations. These cyanobacteria might have a greater ability to maintain their higher growth rate than the other alkaliphiles, particularly when they face a wide dynamic range in HCO_3_^−^ availability, i.e. during cyanobacterial bloom, by utilizing BicA at high HCO_3_^−^ and SbtA at low HCO_3_^−^ conditions.

In regards to BCT1, it has been reported as an inducible transporter under C_i_ limitation [12], [78] and high-light stress [79]. This transporter is the medium substrate affinity class (K_m_ = 10–15 μM) [18]. Comparative genome analysis showed that this transporter is found only in freshwater β-cyanobacteria and four strains of the moderate alkali-thermophiles, but not in other moderate alkali-mesophiles, strong alkaliphiles, and the marine cyanobacteria (Table 2 and Fig. 5). Since the ATP binding cassette transporter BCT1 helps facilitating bicarbonate transportation, its presence is crucial for cyanobacteria living in freshwater where the contents of inorganic carbon and ions are extremely low. However, the reason why BCT1 is required in all the moderate alkali-thermophiles is not obvious. It is speculated that high temperature might limit the solubility of inorganic carbon and ions in such environment. This is supported by Kamennaya et al. [80] who reported that some inorganic carbons can form insoluble carbonate, of which its solubility is decreased with increasing temperature. The lack of BCT1 in all of the alkali-mesophiles and highly-alkaliphiles indicates the unnecessity of this transporter and the adaptation of such cyanobacterial groups residing in the saline alkaline environments with enriched carbonate ion.

Finally, diversity of CAs was observed among freshwater, marine, and alkaliphilic β-cyanobacteria. While carboxysomal γ-CA, CcmM, was observed in all cyanobacteria, β-CA, CcaA, was only found in some cyanobacteria (Fig. 5). Nine out of the twelve investigated alkaliphilic strains were found with the absence of CcaA. The reason why the strains living in the high pH conditions tend to lose CcaA is still unclear. On the contrary, the existence of CcmM in all studied strains is not surprising given the role it plays in β-carboxysomes. The evolution of CcaA and CcmM within the carboxysomes of these alkaliphiles remains to be evaluated. Thus far, it has been believed that CcmM not only functions as a shell protein for carboxysome but also as CA activity for the strains lacking CcaA. Peña et al. in 2010 [73] reported the CA activity in *Thermosynechococcus elongatus* BP-1 possessing only CcmM, and later de Araujo et al. in 2014 [74] suggested that activity of γ-CA might be regulated by RbcS-like domains in CcmM. Recently, Kupriyanova et al. 2016 [48] has attempted to reveal the function of CA in the haloalkaliphilic cyanobacterium *Microcoleus* sp. IPPAS B-353 possessing both CcaA and CcmM by using Western blotting and CA activity assay. Results showed that CcaA functions as an active non-carboxysomal CA, whereas CcmM did not have CA activity in this alkaliphilic cyanobacterium.

## 5 Conclusion

The molecular components of CCM in 12 alkaliphilic cyanobacteria were identified in this study. The diversity and adaptability in the C_i_ uptake systems and CAs of such cyanobacterial species were observed. Remarkably, the existence of HCO_3_^−^ transporters greatly differs among the alkaliphiles. It seems likely that alkaliphilic cyanobacteria tend to modify their CCM components in response to the environmental influence (moderately to strongly alkaline habitat). These reflect the capability of the strains to survive and establish competitive growth by using different C_i_ uptake strategies at changes of CO_2_ and HCO_3_^−^ levels. This insight into the CCM components of the alkaliphiles provides fundamental knowledge for further research towards improvement of photosynthetic CO_2_ fixation in some economically important cyanobacterial strains and crops.

The following is the supplementary data related to this article.

Supplementary File Detailed annotation of CCM genes and protein of 12 studied alkaliphilic cyanobacteria. Supplementary File

## Conflicts of Interest

The authors declare no conflicts of interest.

## Competing Interests

The authors declare that they have no competing interests.
